# The twin epidemic in infertility care – Why do we persist in transferring too many embryos?

**Published:** 2016-12

**Authors:** W Ombelet

**Affiliations:** Editor-in-Chief; Genk Institute for Fertility Technology, ZOL Hospitals, Schiepse Bos 6, 3600 Genk, Belgium.

**Keywords:** Assisted reproduction, economics, ICSI, IVF, IUI, multiple pregnancy, obstetrics, perinatal risks

## Abstract

The epidemic of iatrogenic multiple births as a result of infertility treatment are responsible for an unacceptable high incidence of maternal, perinatal and childhood morbidity and mortality. Healthcare costs due to infertility therapy are too high and this may lead to social and political concern.

The introduction of single embryo transfer (SET) was a real breakthrough, but was only accepted in most European countries and Japan. The United States, Latin America and most developing countries still have high multiple pregnancy rates. The most common argument for not performing SET are the high costs associated with ART procedures. Competition between ART centres to achieve and publish the highest success rates is another major factor.

But things have changed: vitrification methods for cryopreservation are responsible for a better survival and increased success rate with frozen-embryo transfer, our knowledge to select the best embryo for SET is increasing and the growing concern of health care providers and governments can be expected in the near future. Infertility specialists are supposed to deliver healthy, preferably singleton babies at the lowest cost. Misuse of science still reveals the dark side of ART in too many centres.

There is enough evidence that reimbursement policies providing accessible ART to infertile couples can decrease the potential harm from multiple pregnancies substantially unless we succeed to provide simplified IVF at affordable prices.

In the late Nineties and the early 2000s some conflict developed between infertility specialists, obstetricians and neonatologists/paediatricians: the increasing incidence of high-risk pregnancies associated with assisted reproductive techniques (ART) was obvious and the NICUs (neonatal intensive care units) were overwhelmed and occupied by babies born after ART, whether IVF- related procedures or due to non-IVF ovarian stimulation treatment.

In 1997, 19 years after the first IVF infant was born, Dunn and MacFarlane (1997) were the first to draw attention to the risks associated with multiple pregnancies as a result of ART. Data for England and Wales showed that between 1975 and 1994 the twin maternity rate increased by 35% and the triplet and high order multiple pregnancy rate had more than trebled, as a consequence of increased use of ovulation induction and multi-embryo transfer in the treatment of subfertility.

Multiple pregnancies associated with infertility treatment are recognized as a major complication and are large contributors to the extreme prematurity and very low-birth-weight population. Due to the epidemic of iatrogenic multiple births, the incidence of maternal, perinatal and childhood morbidity and mortality has increased worldwide. Subsequently a much higher healthcare cost of infertility therapy can be expected which may lead to social and political concern.

Reducing the number of embryos transferred was the first option to reduce multiple pregnancies. In 2003 the Belgian government decided to reimburse 6 IVF/ICSI cycles in a lifetime, provided a strategy was followed whereby a decreasing number of embryos are transferred in order to reduce the twin pregnancy rate (Ombelet et al., 2005). As a result of the Belgian strategy the twin pregnancy rate has dropped from 26.5 % in 2002 to 9.7 % in 2014 (BELRAP, 2016). Although Belgian infertility specialists were aware of the twin epidemic and knew about the possible advantages of single embryo transfer (SET) many years before the reimbursement policy was launched in 2003, a significant drop of twins could only be observed from 2004 on, which means that only a financial incentive made the difference (Fig. 1). The same accounted for most countries that followed the Belgian example. One of the exceptions is Japan. In this country ART practices seems to be regulated by the rules and moral policy of a society without any stringent regulation. It is surprising that Japan obtained an 86.2 % of SET in 2012, compared with 59.6 % in Belgium (BELRAP, 2016; Takeshima et al., 2016). Also the Scandinavian countries score very well with a 5.2 %, 5.6 % and 7.5 % twin rate in Iceland, Sweden and Finland respectively (Calhaz-Jorge et al., 2016).

**Fig. 1:**
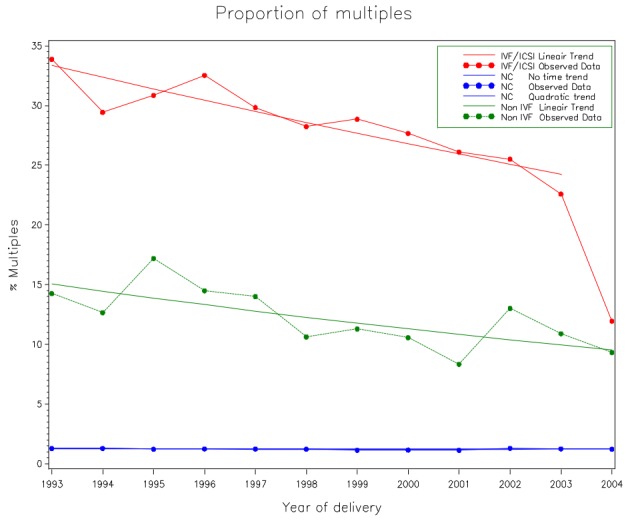
— The evolution over time of the probability of multiple deliveries after IVF/ICSI, non-IVF OS and natural conception in Flanders between 1993 and 2004. The OR of the multiple pregnancy rates for the different groups in 2003 versus 2004 was estimated based on a logistic regression model with an unstructured time effect. The OR equalled 2.151 with 95% confidence interval [1.75, 2.65], p < 0.0001. Using the data from 1993 to 2003 it turns out that the logit of the probability of a twin or triplet delivery after IVF/ICSI was linearly decreasing over time. The OR equalled 0.956 with a 95% confidence interval [0.944, 0.970], p<0.0001 (OR = odds ratio, Non IVF = ovarian stimulation group without IVF/ICSI, NC = natural conception).

Pressure to achieve higher pregnancy rates in infertility treatment still result in unacceptable high multiple pregnancy rates in many countries. For 2012, the European Society of Human Reproduction and Embryology (ESHRE) reported a 17.3 % and 12.2 % twin delivery rate after fresh embryo and frozen embryo transfer respectively (Calhaz-Jorge et al., 2016). In 2013 multiple births still occurred in 20.3 % of IVF/ICSI cycles among women younger than 35 years in the United States (Hornstein, 2016) and in Latin American the prevalence of twins and triplets was still as high as 20.7 % and 1.09 % in fresh autologous IVF/ICSI cycles (Zegers- Hochschild et al., 2016). US data show that twin births nearly doubled over the last three decades to 1 in 30 babies born in the United States in 2009, from 1 in every 53 babies in 1980.

Recent reports indicate that the perinatal outcome of children born after ART improve over time mainly explained by less multiples because of the increased use of elective single-embryo transfer (Henningsen and Pinborg, 2014; Henningsen et al., 2015). A refinement of both clinical and laboratory skills during the past three decades can be the explanation, the most important being the better selection of the embryo with the highest implantation potential through blastocyst culturing, metabolomics, time- lapse imaging and PGS (preimplantation genetic screening). Above this, frozen embryo transfer became more popular not only due to the better survival rates obtained with the vitrification technology, but also because of reports indicating that SET of frozen/thawed embryos may be beneficial to the offspring due to the possible advantages of a nonstimulated endometrium (Morimoto, 2016). Other possible factors are the increased use of soft catheters and ultrasound guidance when the embryo transfer is performed and the better understanding of the importance of the uterine cavity thanks to better imaging techniques and high quality hysteroscopic surgery.

There is enough evidence that a SET policy will increase the safety of ART children without decreasing success rates significantly.

But the question remains: why do many infertility specialists still succeed to transfer multiple embryos. The most striking example is the high incidence of DET and subsequently twin pregnancies in oocyte donation programmes. They deal with young oocyte donors with high quality embryos. A twin pregnancy rate of almost 50 % can be expected after DET in egg donation programmes (Clua et al., 2015). Nevertheless, when looking at the websites of many oocyte donor programmes in Spain we still observe unexpectedly high multiple pregnancy rates due to multiple embryo transfer. They argue that oocyte donation is expensive and patients rather prefer to have DET instead of SET to increase the possibility to become pregnant in that specific cycle and also because cryopreservation of embryos is expensive as well. I doubt whether the patients are well informed about the consequences of multiple pregnancies in all centres with a low SET performance. The same counts for the US. The average cost of an IVF cycle in the United States is approximately between 10000 and 15000 $ (Hornstein, 2016). One can imagine that patients want to maximize the success rate and ask for multiple embryo transfer, whatever the consequences are.

Therefore I believe that funding arrangements and/or reimbursement policies that minimize out-of-pocket expenses will not only maximize equity of access but they will also minimize the potential harm from multiple pregnancies. An alternative road is to make use of simplified methods to make IVF less costly but equally effective (Ombelet et al., 2014). The suggested strategies will surely result in a higher proportion of single healthy children - the ultimate goal of infertility treatment.
